# Active Image-Assisted Food Records in Comparison to Regular Food Records: A Validation Study against Doubly Labeled Water in 12-Month-Old Infants

**DOI:** 10.3390/nu10121904

**Published:** 2018-12-04

**Authors:** Ulrica Johansson, Michelle Venables, Inger Öhlund, Torbjörn Lind

**Affiliations:** 1 Department of Clinical Sciences, Pediatrics, Umeå University, SE 901 85 Umeå, Sweden; inger.ohlund@umu.se (I.Ö.); torbjorn.lind@umu.se (T.L.); 2 MRC Elsie Widdowson Laboratory, Cambridge CB I 9NL, UK; Michelle.Venables@mrc-epid.cam.ac.uk

**Keywords:** energy intake, dietary assessment, image-assisted method, infant, food record, doubly labeled water

## Abstract

Overreporting of dietary intake in infants is a problem when using food records (FR), distorting possible relationships between diet and health outcomes. Image-assisted dietary assessment may improve the accuracy, but to date, evaluation in the pediatric setting is limited. The aim of the study was to compare macronutrient and energy intake by using an active image-assisted five-day FR against a regular five-day FR, and to validate image-assistance with total energy expenditure (TEE), was measured using doubly labeled water. Participants in this validation study were 22 healthy infants randomly selected from the control group of a larger, randomized intervention trial. The parents reported the infants’ dietary intake, and supplied images of main course meals taken from standardized flat-surfaced plates before and after eating episodes. Energy and nutrient intakes were calculated separately using regular FR and image-assisted FRs. The mean (± standard deviations) energy intake (EI) was 3902 ± 476 kJ/day from the regular FR, and 3905 ± 476 kJ/day from the FR using active image-assistance. The mean EI from main-course meals when image-assistance was used did not differ (1.7 ± 55 kJ, *p* = 0.89) compared to regular FRs nor did the intake of macronutrients. Compared to TEE, image-assisted FR overestimated EI by 10%. Without validation, commercially available software to aid in the volume estimations, food item identification, and automation of the image processing, image-assisted methods remain a more costly and burdensome alternative to regular FRs in infants. The image-assisted method did, however, identify leftovers better than did regular FR, where such information is usually not readily available.

## 1. Introduction

Dietary assessments from food records (FR) are commonly used to assess children’s food and nutrient intake, and possible relationships between dietary intake and health outcomes [1]. In infants and young children, parents are asked to record everything that the child has eaten and drunk during a predefined time period [2]. However, achieving accurate and reliable dietary intake data can be difficult and demanding: for the parents, the process may be tedious and time consuming [3], and for the clinician or researcher, the generated data may be subject to bias, making interpretation difficult [4]. Meals with complex content, such as main course meals with several ingredients, are challenging to remember, record, and to determine the amounts of the various ingredients [5]. In young children, food records tend to overestimate energy intake, e.g., parents may misreport the child’s intake by failing to omit food leftovers and spillage from the FRs [4,5,6]. In order to better understand the complex relationships between diet and health in young children, it is important to develop dietary assessment methods with higher accuracy and precision [7]. 

Mobile phone applications and cameras have been shown to improve self-reported dietary intake, and they have likewise increased participants’ user satisfaction, compared to conventional methods [8]. Moreover, information obtained from images seems to reduce random and measurement errors for energy intake (EI), especially when it comes from complex and diverse foods. In adults, EI is often underestimated, but this can be corrected with image-assisted dietary assessments [9]. This was also found among overweight and obese children by using digital camera FRs [10]. Previous research in pre-school children has shown that EI assessment using images was not significantly different, compared to measuring total energy expenditure (TEE) with doubly labeled water (DLW) [11]. However, no study to date has investigated methods of active image-assisted FRs in infants [9,12]. 

The aim of this study was to compare total and main course meal energy and macronutrient intake in 12-month-old, healthy infants using an active image-assisted five-day FR against a regular five-day FR, and to validate the total energy intake measured with the image-assisted food record method against TEE using DLW. 

## 2. Materials and Methods 

### 2.1. Participants and Study Design 

The infants in the present study were taking part in an optimized complementary feeding study (OTIS; ClinicalTrials.gov registration number NCT02634749, (*n* = 250) among 4–6 months old, healthy, full-term infants in Umeå, Sweden, measuring the effects of different complementary diets on various health outcomes and food acceptance. In the present validation study, all infants (*n* = 27) belonging to the control group in the OTIS trial from September 2016 until July 2017 were selected at 12 months of age. In the control arm of the study (*n* = 125), the participants were advised to follow the current, Swedish dietary recommendations, but they were otherwise not subject to any intervention [13]. In the present study, as well as in the larger OTIS trial, the inclusion criteria were healthy, singleton infants, 4–6 months of age, born after >37 weeks of gestation and birth weight > 2500 g, living in Umeå municipality. The exclusion criteria were infants with chronic illnesses, iron deficiency, or any other biochemical abnormality, or infants been having started feeding with complementary foods at the time of recruitment. 

### 2.2. Anthropometry 

Within two weeks of the participants’ 12-month birthday, the infants were invited to the Pediatric research facility at Umeå University Hospital for information on the study procedures, measurements and administration of DLW. Anthropometric data were collected according to standardized procedures [14]: nude weight was measured to the nearest 5 g using electronic scales (Seca 727, Seca, Hamburg, Germany), recumbent length was measured to the nearest 0.1 cm using an infantometer (Seca 416, Seca, Hamburg, Germany), and the head circumference was measured to the nearest 0.1 cm by using a non-stretchable measuring tape (Seca 212, Seca, Hamburg, Germany). 

### 2.3. Doubly Labeled Water 

On the same day as the anthropometrical measurements, a pre-dose urine sample was collected by placing an absorbent pad (Bastos Viegas, Penafiel, Portugal) in the diaper of the infant. Each infant was then given an oral weighed dose of DLW consisting of 100 mg/kg ^2^H_2_O and 280 mg/kg H_2_^18^O. Post-dose urine samples were collected at home once daily for 10 consecutive days, with dates and times recorded for all samples by using absorbent pads as described above, omitting the first urine portion of the day. The first post-dose urine sample was collected approximately 24 h after the DLW dose was given and the subsequent pads were collected once daily after that. The parents were asked to remove the pad once it was wet from urine. Each collected pad was stored at −18 °C. The pads were then taken to the Pediatric research facility at Umeå University Hospital and thawed, and the urine content was extracted using a press, collecting the urine in glass bottles. The glass bottles were stored at −20 °C until transportation to MRC Elsie Widdowson Laboratory, Cambridge, UK for analysis. 

### 2.4. Water Isotope Analysis

Urine samples were prepared for ^18^O enrichment using the CO_2_ equilibration method [15]. The samples were then analyzed using a continuous flow isotope ratio mass spectrometer (IRMS) (AP2003, Analytical Precision Ltd., Northwich, Cheshire, UK). For ^2^H, the samples were analyzed using a continuous-flow IRMS (Sercon, ABCA-Hydra 20–22, Sercon Ltd., Crewe, UK). All samples were measured alongside secondary reference standards previously calibrated against the primary international standards Vienna-Standard Mean Ocean Water (vSMOW) and Vienna-Standard Light Antarctic Precipitate (vSLAP) (International Atomic Energy Agency, Vienna, Austria). Sample enrichments were corrected for interference according to Craig [16], and expressed relative to vSMOW. Analytical precisions (SD) were better than ±0.4 ppm for ^18^O and ± 1.3 ppm for ^2^H. The rate of CO_2_ production ($R_{CO_{2}}$) was calculated according to Schoeller et al. [17], $R_{CO_{2}}$ was then converted to TEE using the equation of Elia and Livesey [18], with the food quotient (FQ) calculated according to Jéquier et al. [19]. From TEE, metabolizable energy (ME) was calculated according to Wells and Davies [20].

### 2.5. Food Record and Dietary Assessment

Parents were asked to record everything that their child ate and drank, including breastmilk and food supplements, e.g., vitamins, using a pre-printed five-day FR. Of these five days, we asked that at least one day was a Saturday or Sunday. The parents started the recording the day after the administration of DLW. Each day, the parents recorded the meal type, time of day, and which foods and drinks the infants were offered, including amounts and brand names. Amounts of foods and drinks were documented using household measures and for bread etc., in slices. Homemade recipes were documented separately, including ingredients, quantities and detailed descriptions of preparation. Unfamiliar dishes were reported in detail with brand name and amounts. Breastmilk was recorded as ‘meals’ (more than five minutes of breastfeeding) or ‘snacks’ (less than five minutes of breastfeeding), estimated as 102 or 25 g of milk, respectively [21,22]. The reported food and drink intake was converted to grams using standardized weights for consumed foods from the Swedish Food Agency Database [23]. To calculate the mean daily EI (kJ/day) and macronutrients sub-classes (g/day) from the five day FR, we used the software Dietist Net Pro (Kost och Näringsdata AB, Bromma, Sweden) and the food composition database (version 17 February 2016) from the Swedish National Food Administration. The database was complemented with special products for infants used in the OTIS study, with nutrient contents analyzed and supplied from Semper AB.

### 2.6. Food Record with an Active Image-Assisted Method

An active image-assisted FR method is a system that captures images, usually photographs, during eating episodes, and is used to enhance or supplement traditional written or electronic FRs [24]. The images provide objective information such as food type, volume, and leftovers, and may even record foods that were forgotten and not reported in the food registration [24]. In this study, we decided to capture two main meals, i.e., the noon (lunch) and late afternoon (dinner) meals, for the image-assisted part. These two meals were expected to represent 30% of the total daily EI, and they included more complex and diverse dishes with a larger amount of ingredients mixed together, which makes assessing the composition and estimating leftovers more challenging [8]. Given the meal frequency of 12-month-old infants and without specific smart phone applications to facilitate this task, we also assumed that the workload for the parents would be too great if they would have to record all of the meals that the child consumed by using the image-assisted method.

**Figure 1:**
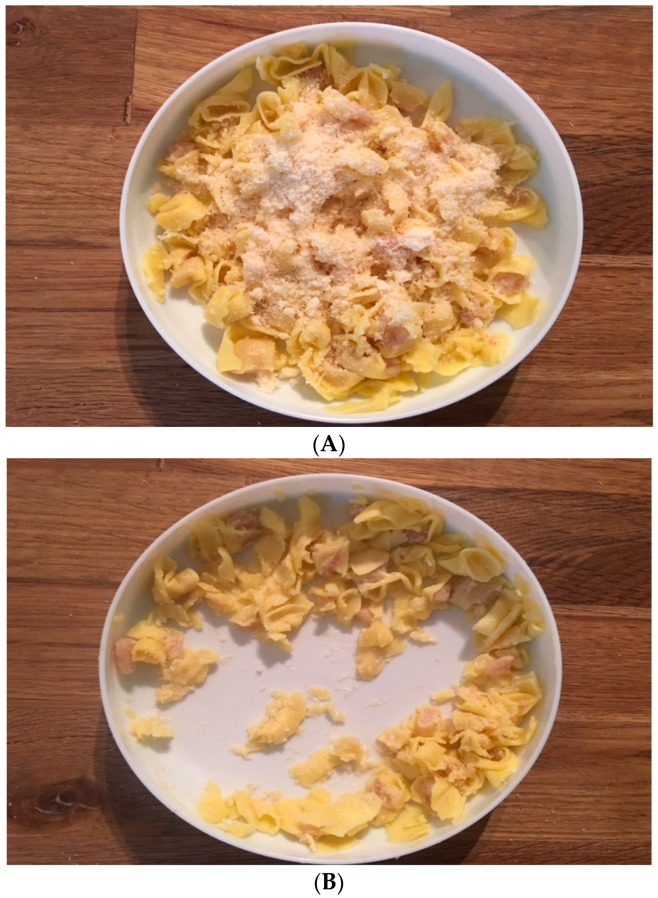
Images of a main course meal with leftovers before (**A**) and after (**B**) an eating session.

During the five-day FR, parents were instructed to serve the two main meals on standardized flat-surfaced plates, which were provided by the researchers to the participants, and then to capture mobile phone images of the plates before and after each meal. The plate served as a reference marker [25]. The written instructions had four examples of main meals served on the standardized flat-surfaced plate before and after intake, with images captured at a 90° angle from a mobile phone camera according to Stumbo (Figure 1) [26]. All 20 images from the 10 main meals during the five-day FR were sent by the parents, usually by directly sharing the images from the participants’ mobile phones to the study e-mail account. The participants used their own mobile phones for the photos. If no images were received within five days, a reminder was sent by e-mail to the participant. First, a trained pediatric dietician calculated the mean, daily energy, and macronutrient intakes from the FRs without access to the images. In a second step, the images of the main meals were made available for the dietician, who analyzed the images, taking food leftovers, spillage, etc., into consideration [25]. To assist the dietician in estimating the food items and food volumes on the plate before and after the meal, the dietician was provided with images of the standardized flat-surfaced plate with different quantities of commonly used baby foods, either from glass jars as used in the study, or home-cooked food comparable to an infant’s normal portion size. These reference images were similar to the images received from the participants [24]. Finally, the initial calculations from the FR were, if needed, adjusted depending on the results of the analyses of the main meal images, for example, subtracting undocumented leftovers and spillages from the initially estimated intake. This generated two sets of data, one from the regular FR, and one record that included the image-adjusted dietary intakes. The latter also contained specific information on leftovers that was unaccounted for in the regular FR. 

### 2.7. Pilot Testing

Before embarking on the present study, we performed an unpublished pilot study to assess the feasibility of using image-assisted FR in infants. Parents of fourteen 8–12 months old, healthy, free-living infants were asked to do a five-day food record in a similar way as in the present study, taking mobile phone images of the main course meal together with a regular FR. During the course of five days, 78 meals were recorded by the participants. When comparing the food records with and without image assistance, we found that 23 meals (29%) had to be adjusted, since the meal images contained additional information to correctly estimate the intake from the meals, and 22 of the 23 adjusted meals were overestimations, i.e., the parents had omitted to exclude foods left over or spilled on or around the plate. This resulted in a mean difference in daily EI between the two estimations of 169 ± 146.4 kJ.

### 2.8. Group Size Calculation

We based the sample size calculation on the pilot study described above. We estimated that 45% of participants would have their FR adjusted when image-assistance was added, and that the difference in EI measured with FRs against TEE with DLW would be 238 ± 193.7 kJ [4]. Given these circumstances, and allowing for a 30% attrition rate, we calculated that we would need 25–30 participants (power 90%, alpha = 0.05) in order to show a significant difference in the measurement error in EI between FRs with and without image-assistance, compared to ME.

### 2.9. Ethical Considerations

The study was approved by the Regional Ethical Review Board at Umeå University, Sweden (dnr 2016-134-32M). 

### 2.10. Statistical Analyses 

Statistical analyses were performed using SPSS 24.0 (SPSS, Chicago, IL, USA). For continuous variables, results are presented as means (± standard deviations, SD or ± 95% confidence intervals, CI) and for categorical variables as numbers and percentages. Normal distribution for continuous variables was assessed with the Shapiro–Wilk test. The energy and macronutrient intake were calculated as kilojoules (kJ) and grams (g) per day, respectively. The significance level was set at *p* < 0.05. Differences between image-assisted FR and regular FR and image-assisted FR and ME were analyzed separately with paired sample *t*-tests. The Bland and Altman method [27] was used to assess the agreement between regular and image-assisted FRs, and between the image-assisted FR and ME calculated from DLW. Reliability between ME and the image-assisted FR method was quantified using a two-way mixed absolute agreement intra-class correlation coefficient (ICC).

## 3. Results 

Of the 27 selected infants, 82% completed the study with a majority being boys (Table 1). Five infants were excluded; three with missing FR information and images, and two infants had insufficient urine samples to allow for the analysis of TEE. 

### 3.1. Energy and Macronutrient Intake 

**Figure 2:**
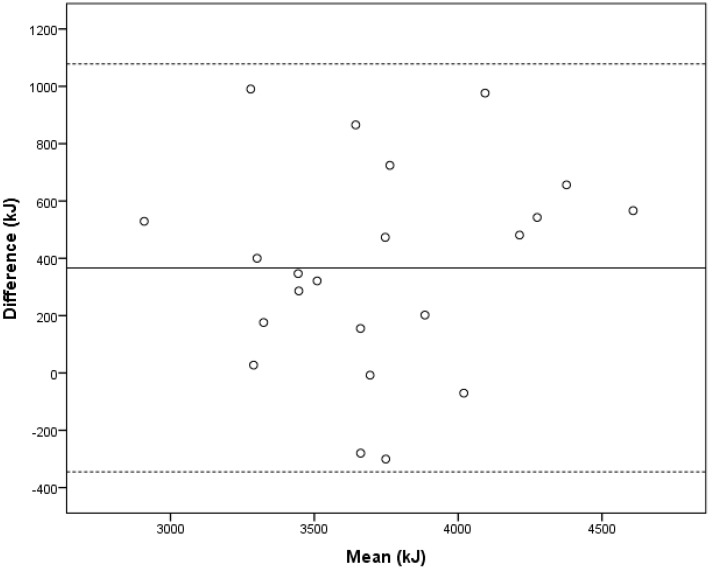
Bland–Altman plot showing the mean versus the difference in energy intake estimated from food records with active image-assistance and metabolizable energy calculated from doubly labeled water in 22 healthy, 12-month-old infants. The *x*-axis shows the mean energy intake (EI) per day (kJ) from FR with image-assistance and metabolizable energy. The solid line (-) shows the mean difference of 366 kJ, and the dashed lines (---) show the 95% limits of agreement (±1.96 SD) of 712 kJ.

Five of the 22 infants were breastfed, usually 2–3 times per day; in the morning, in the evening, and/or at night. None of the infants were breastfed at the time of any of the main course meals. The average number of meals per day was 7.1 ± 1.1, and image-assistance was used in 29% of these meals. Mean EI and macronutrient intakes, both overall and from the main course meal, were normally distributed. Overall, these intakes were not significantly different between the regular FRs and the image-assisted FRs (Table 2). In particular, EI from the main course meals were a mix of equal numbers of over- and underestimated meals (Table 3) and therefore the errors were balanced out and had no effect on the average energy or macronutrient intake (Table 2). Average ME, calculated from DLW was 3538 ± 428 kJ/day. Bland–Altman plots were used to assess agreement between image-assisted FRs and ME. The mean bias between the methods was 366 kJ/day, with limits of agreement of ±712 kJ/day (Figure 2). There was no significant association between the mean and the difference of EI and ME (*p* = 0.53), indicating no systematic bias across the different levels of EI. The intra-class correlation (ICC) coefficient was 0.81, indicating high reliability between the two methods. 

When estimating EI from only the main course meals representing 35% of the total daily EI, i.e., when the image-assisted method was used, there was no statistically significant difference between the image-assisted and regular FRs, and no significant differences for any of the macronutrients (Table 2).

### 3.2. Main Course Meals with an Active Image-Assisted Method

In the five-day FR, 220 main course meals were recorded. Of these, 210 meals (96%) were assessed with both regular FRs, and the active image-assisted method. Ten meals (4%) were excluded because of missing images after the eating episode (Table 3). For the majority of meals, the dietician did no adjustment of the amounts of food consumed from that particular meal after taking the meal images into account. However, for a third of the meals, some adjustments were made. Out of these adjusted meals, about half were underestimations on the part of the regular FR, and leftovers were more common, compared to no leftovers. Of the 22 infants, 17 (77%) had at least one main meal adjusted by the active image-assisted method. The average number of main meals with leftovers over five days were 5.8 ± 3.3 per infant. Three infants had leftovers from all 10 meals, and one infant had no leftovers from any of the eating occasions. 

## 4. Discussion 

Previous studies in infants have shown the non-random error to be 5–15% when comparing energy expenditure, measured with doubly labeled water to recorded dietary intake [1]. Such bias increases the risk of type II-error and diminishes the power of the study. Image-assistance, where FRs are complemented with images before and after eating sessions is one possibility to reduce this bias [9].

In the present study, comparing total EI in regular and image-assisted FRs, the difference between methods was only 3.9 kJ and not statistically different. The reliability between image-assisted FR and ME, and the golden standard to assess EI, using ICC was excellent. The bias of 366 kJ means that image-assisted FRs overestimated the EI compared to ME by 10%. This bias was higher but the limits of agreement were narrower than reported in similar studies [4,11]. Previous studies have shown that image assistance has been successful in reducing underreporting, but to our knowledge no study has used the technique in settings when over reporting is an issue, as was the case in the present study [9,10]. 

Unaccounted leftovers, i.e., the parts of the meal that are left on the plate or that are lost due to spillage, are possible sources of systematic error if they are not subtracted from the estimated intake. In the present study, 60% of the meals that were recorded with active image assistance showed leftovers. However, in more than half of these eating episodes, the parents correctly modified the FR to include the leftovers, and in the other half, where the research dietician did adjust the recording, it was equally common for the dietician to increase the recording as it was to decrease the estimated EI from that meal. In our earlier pilot study, we found a similar proportion of meals (23/78 meals in the pilot vs 69/210 meals in the present study) had been adjusted, but the regular FR overestimated the energy intake by 169 kJ per day, compared to the image-assisted method. Also, in the pilot, the majority of the adjusted meals (22/23) were overestimations. In the present study, underestimations were equally common to overestimations. A possible explanation to this discrepancy may be that the participants in the present study, being part of a large trial, were more experienced in completing food recordings, this being their third in six months compared to the participants in the pilot. We speculate that greater experience explains some of the lower bias in the regular FRs, compared to the image-assisted method found in our study [28,29]. We do not know to what extent the parents in the present study used the images to corroborate their recordings, but we did notice that the parents were skilled at allowing for leftovers in the FRs, which is indicated by the fact that half of the meals where there were leftovers had been already adjusted by the parent. From the present study, we have no information on the amount of leftovers or spillage from the eating episodes where image assistance was not used. We can only speculate that the overestimation of EI compared to ME can be found in unaccounted leftovers, from for example breakfast and snacks. From the FRs, we know that these meals contained large amounts of energy dense foods, such as porridge and milk cereal drinks. Another possible error could have been the preparation of the porridge and milk cereals, where adding too much water could have made the meals more diluted, reducing the actual EI compared to the recorded EI.

The overall energy and macronutrient intake, and its variations, were similar to other studies in the same age group [30,31,32]. Also, the parents adhered well to submitting the FRs and images, with less than 5% of the main meals having missing images. Breast milk intakes were estimated from feeding episodes and not by direct observation, i.e., test weighing. On average, breast milk contributed to <6% of the mean total daily energy intake. The energy and nutrient content of the products used in the present study (porridge, formula, milk cereal drink, baby food in glass jars) were based on high quality analyzed data supplied from Semper AB.

A strength of the study was that all parents used the same reference marker [25], i.e., the plate estimated the true area of the food portions, and we omitted images of meals, from which other types of plates were used. Also, the same dietician managed all FRs, calculated the dietary intake, and assessed the images, but was blinded as to the outcome, and did not participate in the final analysis, i.e., when the regular FRs was compared to the image-assisted FRs. In the DLW analyses, we used the more accurate FQ [19], instead of the generic RQ suggested by Schoeller et al. [17]. The proportion of parents with university education was higher than people of the same age and gender in the general, Swedish population, and higher than that reported in a recent iron supplementation study in the same geographical area [33]. Despite this, we believe that it is possible to generalize the results to other populations as well.

A limitation in the study was that we did not use the image-assisted method for all meals and snacks. Our hypothesis was to focus on the main meals, i.e., lunch and dinner, which are more complex and diverse in terms of ingredients and nutrient value, and to leave breakfast and snacks, which in this age are less diverse. It is likely that unaccounted leftovers and spillage from energy- and nutrient-dense foods, such as milk cereal drinks or baby porridge, could have been identified through image-assistance. However, we believe that the task of providing before and after photos of every meal and snack, considering the frequency of feedings, including night meals in this age group would have been almost insurmountable for both the parents and researchers. 

Variations among different mobile cameras, ambient light conditions, etc., may have contributed to some of the subjectivity of the image analysis [34]. All images were taken at 90° to the plate, which is the most favorable for capturing which ingredients the meal contained. However, to optimize volume calculations, another photo at 45° would have been preferable [9,24,35]. To improve the quality of the images, and to aid in the volume estimation, some kind of mobile phone application would have been desirable, but to the best of our knowledge no such product validated for use in infants is commercially available [34].

## 5. Conclusions

In conclusion, in 12-month-old infants, the image-assisted method identifies leftovers better than regular FR, where such information is usually not readily available, and it may thereby improve the accuracy of EI and macronutrients. But as seen in this study, parents with earlier experience of food recording were, in many cases, capable of including leftovers in their records, reducing this source of systematic bias. It is possible that this compensation was facilitated by the availability of the images themselves. Also, FRs with or without image-assistance overestimate EI compared to ME. With these caveats, and without validated commercially available software to aid in the volume estimations, food item identification, and automation of the image processing, the image-assisted method remains more costly and burdensome, but possibly a more accurate alternative to regular FRs in infants [34]. In future validation studies, technical solutions for smartphones are required to better identify food items and food volumes from images. Such future software applications would make it possible to estimate more cost-effectively the entire energy intake in infants. Future research should also include training sessions, both for the participants using the technique, and for professionals involved in the dietary assessment with images [36].

## Figures and Tables

**Table 1:** Anthropometric and demographic data of the study infants (*n* = 22) and their parents.

	**Mean ± SD**
Age infants (months)	11.9 ± 0.34
Body weight (kg)	10.5 ± 1.2
Body length (cm)	76.9 ± 3.1
Head circumference (cm)	47.1 ± 0.9
Mothers age (year)	31 ± 5.1
Fathers age (year)	32 ± 5.3
	***n* (%)**
Girls/boys	6 (27)/16(73)
Breastfeeding at 12 mo	5 (23)
≥1 sibling	10 (45)
Education level Mother	
Elementary school	1 (4.5)
High school	5 (22.7)
University	16 (72.7)
Education level Father	
Elementary school	1 (4.5)
High school	7 (31.8)
University	14 (63.6)
Born in Sweden	
Infant	22 (100)
Mother	21 (95.5)
Father	17 (77.3)

SD: standard deviations

**Table 2:** Total daily energy and macronutrient intake, and the daily energy and macronutrient intake from the main course meals (lunch and dinner combined), estimated by regular five-day food records, and food records with image-assistance in the study infants (*n* = 22).

	Food Record ^1^	Food Record with Image-Assistance ^1^	Difference ^1^	*p* for Difference ^2^
Total intake				
Energy (kJ)	3901 ± 476	3905 ± 476	3.9 ± 48.0	0.71
Protein (g)	29.8 ± 5.7	30.1 ± 6.0	0.2 ± 1.1	0.30
Fat (g)	35.4 ± 6.7	35.4 ± 6.5	0.0 ± 0.6	0.89
Carbohydrate (g)	118.3 ± 17.7	118.4 ± 17.2	0.1 ± 1.5	0.80
Main course meals				
Energy (kJ)	1348 ± 388	1350 ± 377	1.7 ± 55	0.89
Protein (g)	13.0 ± 3.9	13.4 ± 4.1	0.4 ± 1.5	0.19
Fat (g)	12.5 ± 4.2	12.7 ± 3.9	0.2 ± 1.3	0.37
Carbohydrate (g)	38.0 ± 13.3	38.5 ± 12.9	0.5 ± 4.2	0.63

^1^ Values are mean ± SD, ^2^ Paired sample *t*-test.

**Table 3:** Numbers of non-adjusted and adjusted meals (corrected by dietician after review of meal images) with or without leftovers assessed with five-day food records with active image-assistance.

Meals (*n* = 210)	Leftovers *n* (%)	No Leftovers *n* (%)	Total *n* (%)
Non-adjusted meals	67 (53)	74 (89)	141 (67)
Adjusted meals	60 (47)	9 (11)	69 (33)
Underestimated ^1^	34 (27)	2 (3)	36 (52)
Overestimated ^2^	26 (20)	7 (8)	33 (48)
Total	127 (60)	83 (40)	210

^1^ Underestimated: the recorded amount of food consumed is less than what is estimated from the meal images. ^2^ Overestimated: the recorded amount of food consumed is more than what is estimated from the meal images.

## Abbreviation

FR	Food record
DLW	Doubly labeled water
EI	Energy intake
ICC	Intra-class correlation coefficient
ME	Metabolizable energy
SD	Standard deviation
TEE	Total energy expenditure
OTIS	Optimized complementary feeding study
